# ZSTK474 Sensitizes Glioblastoma to Temozolomide by Blocking Homologous Recombination Repair

**DOI:** 10.1155/2022/8568528

**Published:** 2022-07-13

**Authors:** Wenhui Jiao, Shan Zhu, Jingrong Shao, Xiaoliang Zhang, Yanglu Xu, Yixuan Zhang, Ran Wang, Yuxu Zhong, Dexin Kong

**Affiliations:** ^1^Tianjin Key Laboratory on Technologies Enabling Development of Clinical Therapeutics and Diagnostics, School of Pharmacy, Tianjin Medical University, Tianjin 300070, China; ^2^Key Laboratory of Immune Microenvironment and Diseases (Ministry of Education), Tianjin Medical University, Tianjin 300070, China; ^3^State Key Laboratory of Toxicology and Medical Countermeasures, Beijing Institute of Pharmacology and Toxicology, Beijing 100850, China

## Abstract

Glioblastoma (GBM) is the most common primary malignant brain tumor in adults. Temozolomide (TMZ) is used as the standard chemotherapeutic agent for GBM but with limited success, and treatment failure is mainly due to tumor resistance. One of the leading causes of TMZ resistance is the upregulation of the DNA repair mechanism. Therefore, targeting the DNA damage response (DDR) is proposed to be an effective strategy to sensitize tumor cells to TMZ. In the present study, we demonstrated that the combined use of the PI3K inhibitor ZSTK474 and TMZ showed synergetic anticancer effects on human GBM cells in vitro and in vivo. The combination treatment led to significantly increased cell apoptosis and DNA double strand breaks (DSBs). In addition, a mechanistic study indicated that TMZ enhanced the homologous recombination (HR) repair efficiency in GBM cells, while ZSTK474 impaired HR repair by blocking the phosphorylation of ATM and the expression of BRCA1/2 and Rad51, thereby sensitizing GBM cells to TMZ. Moreover, TMZ activated the PI3K signaling pathway through upregulation of the PI3K catalytic subunits p110*α* and p110*β* and the phosphorylation of Akt. Meanwhile, ZSTK474 blocked the activity of the PI3K/Akt pathway. Taken together, our findings suggested that the combination of ZSTK474 and TMZ might be a potential therapeutic option for GBM.

## 1. Introduction

Glioblastoma (GBM) in adults is the most common and aggressive grade IV brain tumor [1]. The current standard of care (SOC) for GBM consists of surgical resection, followed by administration of adjuvant chemotherapeutic agent temozolomide (TMZ) and radiation therapy [2]. However, even after aggressive treatment, the prognosis of GBM remains poor, with a median survival time of 14.6 months, and the 5-year survival rate after diagnosis is less than 10% [3]. Although TMZ is the most commonly used and first-line clinical chemotherapy drug for GBM, the combination of TMZ and radiation only modestly improves progression-free survival. Primary or acquired resistance to TMZ is the main reason for the failure of TMZ chemotherapy [4]. Since current treatment options for GBM are limited, an effective chemosensitizing strategy that enhances the efficacy of TMZ is urgently needed.

The phosphoinositide 3-kinase (PI3K)/AKT signaling pathway plays a vital role in cellular processes involved in regulating tumor proliferation, survival, metabolism, metastasis, and chemoresistance [5]. GBM displays aberrant activation of the PI3K pathway, which occurs due to a variety of mechanisms, including overexpression of the EGF receptor, activating mutations of the PIK3CA or PIK3R1 gene, or loss of the negative pathway regulator PTEN [6]. Therefore, direct pharmacologic inhibition of the PI3K/AKT signaling pathway has emerged as a potential strategy for this disease. Currently, PI3K inhibitors used as a single agent or in combination with other therapies are being tested in a number of clinical trials [7, 8]. The pan-PI3K inhibitors BKM120 and PX-866 exhibit better anti-GBM efficiency without obvious side effects [9, 10]. On the other hand, TMZ exerts anticancer activity primarily via the formation of O_6_-methylguanine (O_6_-meG) and subsequent O_6_-meG G:T mispairing, which lead to DNA strand breaks and cell death if left unrepaired [11, 12]. Abrogating the DNA damage response (DDR) to DNA breaks is an attractive combination treatment to sensitize GBM to TMZ [13, 14]. The PI3K pathway has also been reported to be involved in DNA replication and genome stability. PI3K blockade can cause DNA damage through nucleoside depletion in BRCA1-deficient triple negative breast cancer models [15]. PI3K was also demonstrated to preserve DNA double strand break (DSB) repair by interacting with the homologous recombination (HR) complex, which is necessary for DNA repair during ionizing radiation [16, 17]. In addition, we previously showed that the PI3K inhibitor BKM120 increased DNA damage and sensitized GBM cells to PARP inhibition [18]. Therefore, we hypothesized that PI3K inhibition would impair the DDR and subsequently sensitize GBM cells to TMZ.

ZSTK474 is a pan class I PI3K inhibitor that we identified using the JFCR39 drug discovery system. We previously reported that ZSTK474 exerted promising antiproliferative activity on multiple cancer cell lines [19]. ZSTK474 is currently in phase I/II clinical trials for the treatment of solid tumors [20]. Moreover, ZSTK474 showed weak affinities for ABC transporters and pronounced antitumor efficacy in an orthotopic brain tumor model [21]. In the present study, we showed that the combined use of the PI3K inhibitor ZSTK474 and TMZ effectively inhibited GBM proliferation in vitro and in vivo. Furthermore, ZSTK474 promoted DNA damage by suppressing the HR repair process and sensitized GBM cells to TMZ. These results suggest PI3K inhibition as a plausible strategy to increase the efficiency of TMZ in GBM therapy.

## 2. Materials and Methods

### 2.1. Reagents and Antibodies

ZSTK474, BYL719, and TGX221 were purchased from Selleck (Danvers, MA, USA). TMZ was purchased from TOPSCIENCE (Shanghai, China). WST-8 reagent was purchased from Beyotime Biotechnology (Shanghai, China). TRIzol reagent was purchased from Life Technologies (Carlsbad, CA, USA). Star Script II First-strand cDNA Synthesis Mix and 2× Real Star Green Fast Mixture were purchased from GenStar (Beijing, China). The Annexin V-FITC/PI Apoptosis Detection Kit was purchased from BD Biosciences (San Jose, CA, USA). Propidium iodide (PI) was purchased from Sigma-Aldrich (St. Louis, MO, US). Antibodies against PI3K-p110*α* (#4255), PI3K-p110*β* (#3011), Akt (#9272), phospho-Akt (Ser473) (#4060), mTOR (#2983), phospho-mTOR (Ser2448) (#5536), c-Myc (#18583), *β*-actin (#4970), NBS1 (#14956), phospho-ATM (Ser1981) (#13050), BRCA1 (#9010), BRCA2 (#10741), RAD51 (#8875), DNA-PKcs (#38168), Ku80 (#2753), Caspase 3 (#9662), and PARP (#9532) were purchased from Cell Signaling Technology (Danvers, MA, USA). Antibodies against XRCC1 (#ab134056) and *γ*-H2AX (#ab229914) were purchased from Abcam (Cambridge, UK). Antibodies against BAX (#sc-7480) and Bcl-2 (#sc-7382) were purchased from Santa Cruz Biotechnology (Santa Cruz, CA, USA). Alexa Fluor 488- (#ab150113-) conjugated secondary antibodies were purchased from Abcam.

### 2.2. Cell Culture

Human GBM cell lines U87 and U251 were obtained from the cell bank of the Chinese Academy of Sciences (Shanghai, China). The SF295 cell line was kindly provided by the National Cancer Institute (NCI, USA). Cells were cultured in DMEM (for U87) or RPMI 1640 (for SF295 and U251) supplemented with 10% fetal bovine serum (Biological Industries, Kibbutz Beit-Haemek, Israel), 100 U/mL penicillin, and 100 *μ*g/mL streptomycin. All cells were cultured in a humidified incubator with an atmosphere containing 5% CO_2_ at 37°C.

### 2.3. Cell Viability Assay

The inhibitory effects of TMZ and ZSTK474 on the proliferation of SF295, U87, and U251 cells were examined by using WST-8 reagent as we previously described [22]. The cells in the logarithmic growth phase were seeded into 96-well plates at a density of 10^4^/ml. The next day, the cells were treated with different concentrations of TMZ and/or ZSTK474 for 72 h. At the end of the treatment, 10 *μ*l of WST-8 was added to each well, and the plate was incubated for 3 h. The absorbance was measured at 450 nm using an iMark microplate reader (Bio Rad, Hercules, CA, USA). Half-maximal inhibitory concentration (IC_50_) values were calculated from cytotoxicity curves using GraphPad Prism 8 Software (San Diego, CA, United States).

### 2.4. Drug Combination Analysis

A synergistic assay was employed to determine whether the combination of ZSTK474 and TMZ would enhance the antiproliferative effect. Briefly, three cell lines were treated for 72 h with ZSTK474 and/or TMZ at a constant concentration. Cell viability was determined using the WST-8 assay. The combination index (CI) was calculated according to Chou and Talalay's equation [23] using CalcuSyn software. Combination indices (CI) were then calculated, with CI < 0.9, 0.9 < CI < 1.1, and CI > 1.1 indicating synergism, additivity, and antagonism, respectively.

### 2.5. Western Blot Analysis

Western blot analysis was performed as described in our previous report [24]. SF295 and U87 cells were seeded into 6-well plates at a density of 10^5^ cells/well. After treatment with ZSTK474 and/or TMZ for 48 h, the cells were lysed for at least 30 min in RIPA buffer with phosphatase inhibitors. Then, the total protein concentration was detected using a BCA Protein Assay kit. The same amount of protein buffer (50 *μ*g) was separated by 10-12% SDS-PAGE gels by electrophoresis and then transferred to a PVDF membrane under a constant current of 220 mA. The membrane was blocked in 5% skimmed milk powder at room temperature for 1 h and then incubated with appropriate primary antibodies at 4°C overnight. After incubation, the membrane was shaken and washed 3 times in TBST solution and incubated with the respective HRP-conjugated secondary antibodies for 1 h at room temperature. The protein band was visualized using an ECL detection kit (Thermo Fisher Scientific, Inc., Carlsbad, CA, USA) and digitized by scanning.

### 2.6. Quantitative Real-Time PCR

qRT-PCR analysis was performed as we previously reported [25]. After treatment with the indicated drugs for 48 h, the total RNA in SF295 and U87 cells was extracted from cells using TRIzol reagent and quantified by a Nanodrop spectrophotometer (Thermo Fisher Scientific, Inc.). Then, cDNA was synthesized from 1 *μ*g of total RNA using StarScript II First-strand cDNA Synthesis Mix (Genstar). The expression levels of the target gene were detected with aliquots of cDNA samples mixed with 2 × RealStar Green Fast Mixture by using qRT-PCR. The results are expressed as the fold change calculated by the △△Ct method relative to the control sample. The ribosomal subunit 18S was used as an endogenous control. Sequences of the PCR primers were as follows: BRCA1 Fw, 5′-GAACGGGCTTGGAAGAAAAT-3′; BRCA1 Rv, 5′-GTTTCACTCTCACACCCAGA-3′; RAD51 Fw 5′-CAGATGCAGCTTGAAGCAAA-3′; RAD51 Rv 5′-TTCTTCACATCGTTGGCATT-3′. PIK3CA Fw, 5′-CGGTGACTGTGTGGGACTTATTGAG-3′; PIK3CA Rv, 5′-TGTAGTGTGTGGCTGTTGAACTGC-3′; PIK3CB Fw, 5′-GAGATTGCAAGCAGTGATAGTG-3′; PIK3CB Rv, 5′-TAATTTTGGCAGTGATTGTGGG-3′; 18S rRNA Rw, 5′-CAGCCACCCGAGATTGAGCA-3′; 18S rRNA Rv, 5′-TAGTAGCGACGGGCGGTGTG-3′.

### 2.7. Flow Cytometric Analysis of Apoptosis

Apoptosis analysis was performed as described in our previous report [22]. Briefly, SF295 and U87 cells were treated with ZSTK474 and/or TMZ for 48 h. After incubation, the cells were centrifuged and resuspended in binding buffer, followed by staining with propidium iodide (PI) and Annexin V-fluorescein isothiocyanate (FITC) in the dark for 15 minutes at room temperature. The proportion of apoptotic cells was detected by using an Accuri C6 flow cytometer (BD Biosciences) and quantified using FlowJo Software (Tristar, Ashland, OR, USA).

### 2.8. Cell Cycle Distribution Analysis

Cell cycle distribution was analyzed by PI labeling after treatment with ZSTK474 and/or TMZ for 48 h as we previously described [22]. Cells were then collected and fixed with 75% ethanol at 4°C for 24 h. Subsequently, the cells were stained with PI staining solution (50 *μ*g/ml) containing RNase (100 *μ*g/ml) for 30 min in the dark. After staining, the samples were run on a BD Accuri C6 flow cytometer (BD Biosciences) to analyze cell cycle distribution.

### 2.9. Alkaline Comet Assay

An alkaline comet assay was performed as we previously reported [25]. After treatment with ZSTK474 and/or TMZ for 48 h, SF295 and U87 cells were mixed with low melting point agarose at a ratio of 1 : 10 and spread onto comet slides. The slides were then incubated at 4°C for 10 min and immersed in lysis buffer for 30 min and in alkaline unwinding solution for 20 min in the dark. Following electrophoresis, the cells were stained with GOLDVIEW I for 10 min. The quantification of tail DNA was analyzed with CASP software (CaspLab, Wroclaw, Poland).

### 2.10. Immunofluorescence Staining

Immunofluorescence staining was performed as we previously reported [18]. SF295 and U87 cells were seeded on coverslips in 12-well plates at a density of 0.8 × 10^5^ cells/ml and treated with ZSTK474 and/or TMZ for 48 h. The plates were washed with PBS and fixed with 4% paraformaldehyde for 15 min, and the cells were blocked with 3% BSA for 30 min. After blocking, the cells were incubated with rabbit anti-*γ*H2AX polyclonal antibody or rabbit anti-RAD51 polyclonal antibody overnight at 4°C. The coverslips were washed and incubated with the appropriate secondary antibody in the dark for 1 h. Nuclei was counterstained with DAPI. Cells were observed using a Zeiss LSM800 confocal microscope.

### 2.11. Heterotopic Nude Mouse Xenograft Model

The animal experiment was performed in the animal experimental center of Tianjin Medical University in accordance with the guidelines of the Declaration of Helsinki and approved by the animal ethical and welfare committee of Tianjin Medical University. Six-week-old male BALB/C nude mice were purchased from the Vital River Laboratory Animal Technology Company (Beijing, China). U87 cells (2 × 10^7^ cells per mouse) were subcutaneously injected into the right lateral flank of the nude mice. When the tumor volume reached approximately 800-1000 mm^3^, the tumor blocks were divided into 2 × 2 × 2 mm^3^ masses and implanted into the right flanks of 20 nude mice. When tumors reached a volume of approximately 50 mm^3^, the animals were randomly divided into four groups. Each group contained 5 mice treated with vehicle, ZSTK474 (10 mg/kg, ip.), TMZ (20 mg/kg, ip.), or ZSTK474 and TMZ (the same doses as the single agent). Tumor size was measured every three or two days until the endpoint, and the tumor volume was calculated using the formula (length × width^2^)/2. At the end of the 14 days, the mice were sacrificed by using excessive pentobarbital sodium, and the tumors were then removed and photographed.

### 2.12. Statistical Analysis

Statistical analyses were performed using GraphPad Prism software 8.0 (San Diego, CA, USA). Data are expressed as the means ± SD. Significant differences among groups were determined using Student's *t*-test. A *P* value < 0.05 was considered statistically significant.

## 3. Results

### 3.1. TMZ Induced DNA Damage, Enhanced DNA Damage Repair, and Increased the Activity of the PI3K/Akt Pathway in Human GBM Cells

**Figure 1:**
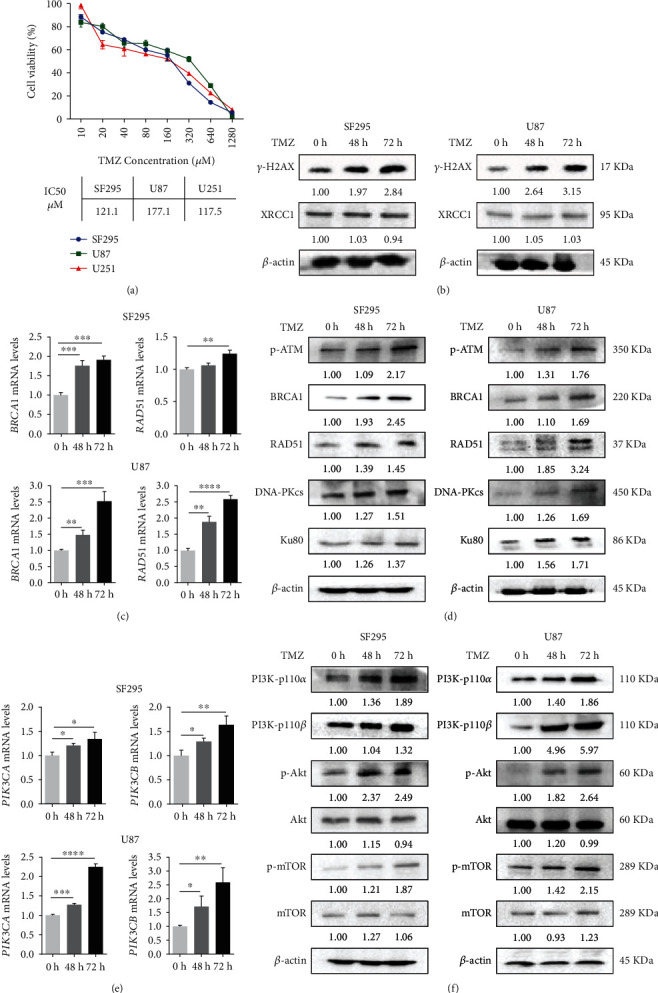
The effect of TMZ on DNA damage and repair regulating factors and key molecules of the PI3K signaling pathway in GBM cells. (a) SF295, U87, and U251 cells were treated with different concentrations of TMZ (10, 20, 40, 80, 160, 320, 640, and 1280 *μ*M) for 72 h. Cell viability was determined by WST-8 assay. (b)–(f) SF295 and U87 cells were treated with TMZ (120 *μ*M and 180 *μ*M) for 48 and 72 h. (b) The cells were then collected for western blotting detection of the DNA SSB marker XRCC1 and the DSB marker *γ*-H2AX. (c) The cells were harvested for qRT-PCR analysis of BRCA1 and RAD51. (d) Western blot analysis of DNA damage repair proteins, including p-ATM, BRCA1, RAD51, DNA-PKcs, and Ku80. (e) qRT-PCR analysis of PIK3CA and PIK3CB. (f) Western blot analysis of key proteins in the PI3K signaling pathway, including PI3K-p110*α*, PI3K-p110*β*, p-Akt, Akt, p-mTOR, and mTOR. For western blotting, *β*-actin was used as a loading control. For qRT-PCR, gene expression was normalized to 18 s rRNA. All data are presented as the mean ± SD (*n* = 3). ∗*P* < 0.05; ∗∗*P* < 0.01; ∗∗∗*P* < 0.001; ∗∗∗∗*P* < 0.0001.

As an alkylating agent, TMZ promotes methylated adduct formation, resulting in DNA damage and tumor cell death [26]. However, this effect will activate the cellular DDR to repair lesions, which causes tumor cells to resist DNA-damaging therapy [4]. To investigate whether TMZ induces DNA repair in GBM cells, we first determined the growth inhibitory effects of TMZ on three human GBM cell lines and then treated the cells with the IC_50_ values of TMZ for 72 h, subsequently examining the change in DNA damage and repair proteins before or after TMZ treatment. As shown in Figure 1(a), TMZ treatment resulted in dose-dependent growth inhibition in three GBM cell lines. TMZ time-dependently increased the expression of *γ*-H2AX, a DNA double-stranded lesion marker, while the expression of XRCC1 (a DNA single-stranded lesion marker) was not affected by TMZ (Figure 1(b)), suggesting that TMZ induced DSBs in GBM SF295 and U87 cells. HR and NHEJ are two major approaches to repair DSBs [14]. We observed that TMZ increased the expression of the HR repair molecules BRCA1 and RAD51 at both the mRNA and protein levels, and the phosphorylation level of ATM was also elevated (Figures 1(c) and 1(d)). In addition, the expression of the NHEJ repair proteins DNA-PKcs and Ku80 also increased after TMZ treatment (Figure 1(d)).

PI3K signaling is known to promote cell proliferation and support DNA repair [27]. PI3K was reported to preserve HR-steady state, and Akt activation could assist DSB repair to promote cell proliferation through upregulation of CDK inhibitor p21 and inhibition of DNA damage [28–30]. Recently, a natural compound, resveratrol was reported to inhibit Akt activation and autophagy flux to impair HR-mediated DSB-repair in breast cancer cells [31]. Therefore, we asked whether TMZ would further enhance the activity of the PI3K/Akt pathway in GBM cells. Western blot analysis showed that TMZ treatment resulted in the upregulation of the PI3K catalytic subunits PI3K-p110*α* and PI3K-p110*β* at the mRNA and protein expression levels. Meanwhile, the phosphorylation of Akt and mTOR, both downstream molecules of PI3K, was also increased in a time-dependent manner. The expression levels of AKT and mTOR were not affected by TMZ treatment (Figures 1(e) and 1(f)). Taken together, these results demonstrated that TMZ caused DSBs and simultaneously activated DNA damage repair and PI3K pathways in GBM cells.

### 3.2. Combined Use of ZSTK474 and TMZ Synergistically Inhibited the Growth of GBM Cells

**Figure 2:**
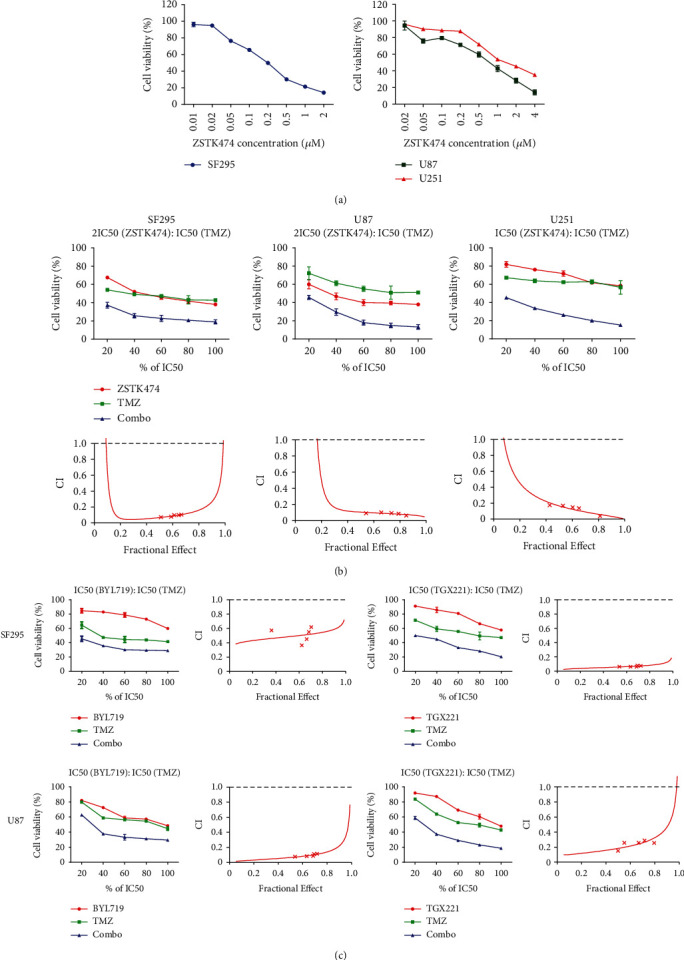
Effect of PI3K inhibitors and TMZ as single agents or in combination on the survival of GBM cells. (a) SF295, U87, and U251 cells were treated with various concentrations of ZSTK474 (0.01, 0.02, 0.05, 0.1, 0.2, 0.5, 1, and 2 *μ*M) for 72 h. Cell viability was determined by WST-8 assay. (b) Three GBM cell lines were incubated with ZSTK474 and TMZ as single agents or in combination (20%, 40%, 60%, 80%, and 100% of 2IC_50ZSTK474_ or IC_50ZSTK474_: IC_50TMZ_) for 72 h. The combination effects were analyzed using CalcuSyn software, and the resulting CI-Fa plots are shown. (c) SF295 and U87 cells were treated with BYL719/TGX221 and TMZ as single agents or in combination (20%, 40%, 60%, 80%, and 100% of IC_50BYL719/TGX221_: IC_50TMZ_) for 72 h. The combination effects were analyzed using CalcuSyn software, and the resulting CI-Fa plots are shown. All data are presented as the mean ± SD (*n* = 3).

We next evaluated whether combination with a PI3K inhibitor would enhance the antitumor activity of TMZ. First, SF295, U87, and U251 cells were treated with the PI3K pan inhibitor ZSTK474 for 72 h, and cell viability was determined by WST-8 assay. As shown in Figure 2(a), ZSTK474 significantly suppressed GBM cell proliferation in a dose-dependent manner, with IC_50_ values of 0.21 *μ*M for SF295 cells, 0.61 *μ*M for U87 cells, and 1.58 *μ*M for U251 cells, respectively. We further treated the three GBM cell lines with increasing concentrations of ZSTK474 and TMZ and analyzed their combinational effect using Chou-Talalay's method. As indicated in Figure 2(b) and Table 1, all the combination index (CI) values were less than 0.9, suggesting the synergistic effect of the combination treatment on all three GBM cell lines. In addition, we used BYL719 and TGX221 to determine whether other PI3K inhibitors could also increase the antitumor effects of TMZ. As shown in Figure 2(c) and Table 1, the CI values were less than 0.9 in all of the treatments in SF295 and U87 cells, suggesting that BYL719 and TGX221 synergistically enhanced the antiproliferative activity of TMZ. Together, these results indicated that inhibition of PI3K signaling and the combined use of TMZ have potential therapeutic efficacy in GBM.

### 3.3. ZSTK474 Synergized with TMZ to Induce Apoptosis in GBM Cells

**Figure 3:**
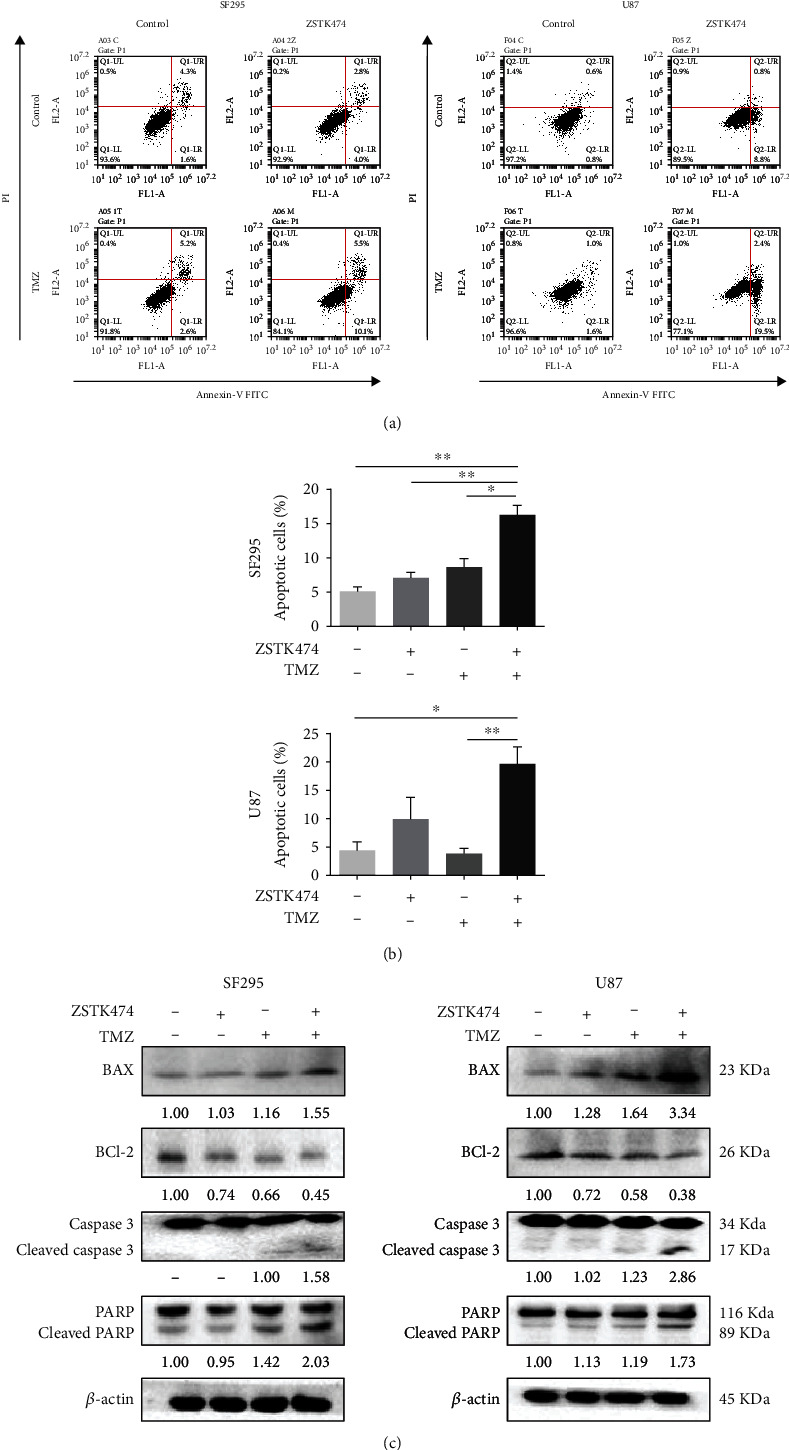
The combined use of ZSTK474 and TMZ synergized to induce apoptosis in GBM cells. (a)–(e) SF295 and U87 cells were treated ZSTK474 (0.4 *μ*M for SF295, 1.2 *μ*M for U87) and TMZ (120 *μ*M for SF295, 180 *μ*M for U87) as single agents or in combination for 48 h (the same concentrations were used in the subsequent in vitro experiments). (a) The percentage of apoptotic cells was detected by Annexin V/PI staining. (b) FACS quantification of the total apoptotic cell population, including Annexin V+/PI− early apoptotic cells and Annexin V+/PI+ late apoptotic cells. (c) The cells were collected for western blotting detection of BAX, Bcl-2, Caspase-3, and PARP. All data are presented as the mean ± SD (*n* = 3). ∗*P* < 0.05; ∗∗*P* < 0.01.

To determine the effect of the combination of ZSTK474 and TMZ on cell death, we conducted an apoptosis assay using Annexin V/PI and FACS analysis in GBM cells. ZSTK474 or TMZ monotherapy each yielded an increase in the apoptotic cell population (early or late apoptotic) to some degree, and the combined treatment led to significantly increased apoptosis in SF295 and U87 cells after 48 h (Figures 3(a) and 3(b)). Consistently, combination treatment also induced a markedly increased level of the proapoptotic protein Bax and a decreased level of the antiapoptotic protein Bcl-2. In addition, the cleavage of caspase 3 and PARP, another signs of apoptosis, was elevated after combination treatment (Figure 3(c)). Next, we investigated the effect of ZSTK474 and TMZ treatment on cell cycle distribution. Treatment with ZSTK474 induced a G1 phase arrest. TMZ alone arrested the cell cycle in G2/M phase, but the effect was attenuated when combined with ZSTK474 (Figure S1).

### 3.4. ZSTK474 Increased TMZ-Induced DSBs in GBM Cells

**Figure 4:**
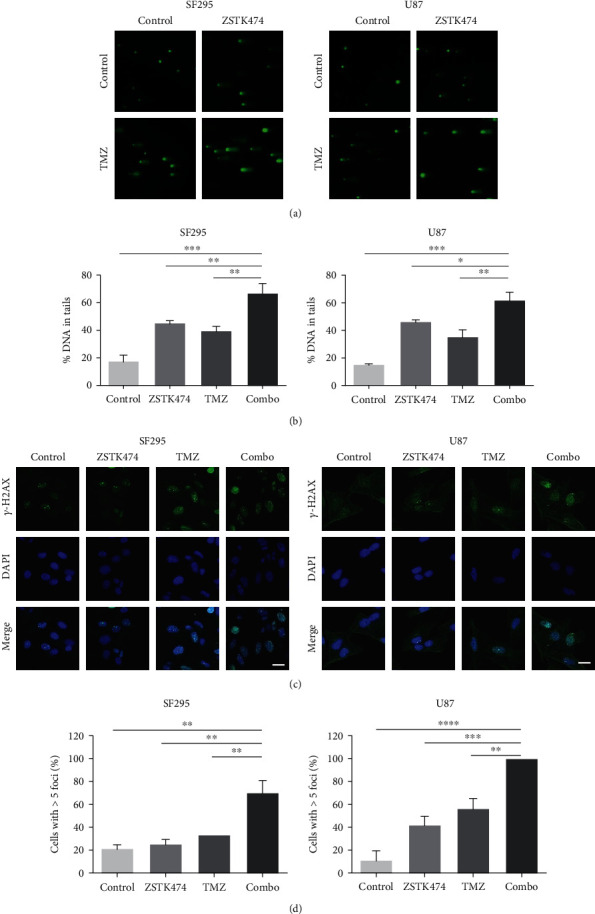
The combined use of ZSTK474 and TMZ cooperated to induce DNA damage in GBM cells. (a)–(d) SF295 and U87 cells were treated with ZSTK474 and TMZ as single agents or in combination for 48 h. (a) An alkaline comet assay was used to detect DNA damage in GBM cells. Comet images ×200 acquired by fluorescence microscopy are shown. (b) The %DNA in tails was quantified to indicate the degree of DNA damage. (c) The formation of *γ*-H2AX foci was assessed using immunofluorescence. Cell nuclei were stained with DAPI. Scale bar, 20 *μ*m. Representative images are shown. (d) Quantification of the number of *γ*-H2AX-positive cells. All data are presented as the mean ± SD (*n* = 3). ∗*P* < 0.05; ∗∗*P* < 0.01; ∗∗∗*P* < 0.001; ∗∗∗∗*P* < 0.0001.

Since DNA damage is a major inducer of apoptosis, we next exposed GBM cells to ZSTK474 and/or TMZ treatment and then subjected them to an alkaline comet assay to evaluate the extent of DNA damage. As shown in Figures 4(a) and 4(b), compared to each single treatment, the combined use of ZSTK474 and TMZ markedly enhanced the tail intensity in SF295 and U87 cells, indicating the production of large numbers of DSBs. Moreover, we examined the nuclear foci of *γ*-H2AX, a biological marker for DSBs, by immunofluorescence staining. Consistent with the comet assay results, the combination treatment significantly increased the number of *γ*-H2AX foci in both cell lines (Figures 4(c) and 4(d)).

### 3.5. Combined Use of ZSTK474 and TMZ Reduced HR Repair Efficiency

**Figure 5:**
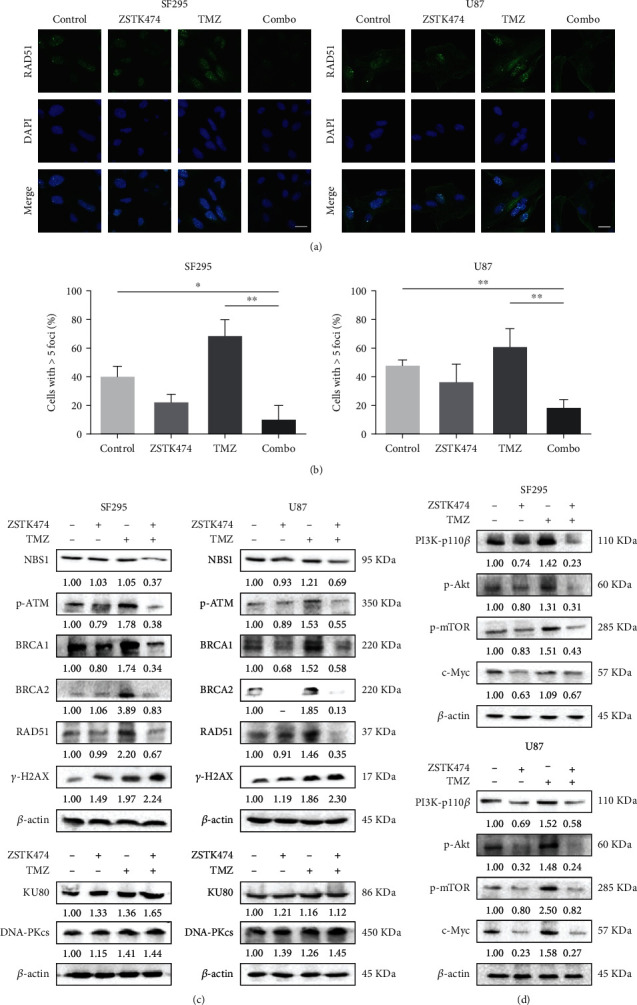
The combined use of ZSTK474 and TMZ reduced HR repair efficiency. (a)–(d) SF295 and U87 cells were treated with ZSTK474 and TMZ as single agents or in combination for 48 h. (a) The formation of RAD51 foci was assessed using immunofluorescence. Cell nuclei were stained with DAPI. Scale bar, 20 *μ*m. Representative images are shown. (b) Quantification of the number of RAD51-positive SF295 and U87 cells. (c) The cells were collected for western blotting detection of NBS1, p-ATM, BRCA1, BRCA2, DNA-PKcs, and Ku80. (d) Western blotting analysis of PI3K-p110*β*, p-Akt, p-mTOR, and c-Myc. All data are presented as the mean ± SD (*n* = 3). ∗*P* < 0.05; ∗∗*P* < 0.01.

To determine the impact of combination treatment on the capability of the cells to repair DSBs, we examined the changes in HR and NHEJ efficiency after drug treatment. First, we conducted immunofluorescence analysis of RAD51, a marker of HR repair capability. As shown in Figures 5(a) and 5(b), the number of RAD51 foci was significantly reduced in GBM cells treated with both drugs compared to TMZ alone, suggesting that the combination treatment could suppress HR efficiency in GBM cells. We then assessed the effect of combination treatment on key molecules in the DNA repair pathway. TMZ increased the expression of the HR repair-related factors NBS1, BRCA1, and BRCA2 and the phosphorylation of ATM, which was reversed by cotreatment with ZSTK474. In contrast, ZSTK474 could not reduce the levels of DNA-PKs and Ku80 in the NHEJ pathway, and the expression of the two proteins even increased after the combination treatment (Figure 5(c)). In addition, we further investigated the effect of the combination treatment on the PI3K signaling pathway. Western blot analysis revealed that ZSTK474 alone or in combination with TMZ resulted in downregulation of PI3K-p110*β*, as well as the downstream effectors phosphorylated Akt, phosphorylated mTOR, and c-Myc, in GBM cells (Figure 5(d)).

### 3.6. Combined Use of ZSTK474 and TMZ Showed Potent Activity in a GBM Subcutaneous Xenograft Model

**Figure 6:**
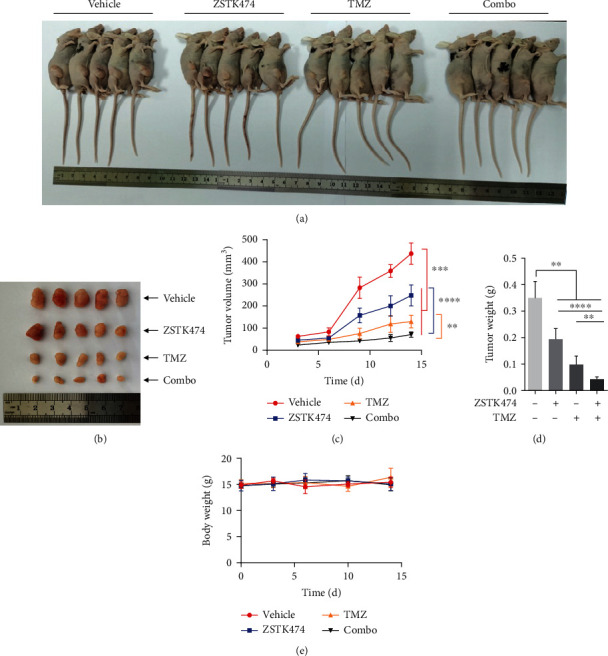
The combined use of ZSTK474 and TMZ exhibited significant antitumor efficacy in vivo. (a)–(d) Nude mice with subcutaneous U87 tumors were treated with vehicle, ZSTK474 (10 mg/kg), TMZ (20 mg/kg), or ZSTK474 combined with TMZ for 14 days. After sacrifice of mice, the images were taken as shown in (a). The tumors were taken and photographed and are shown in (b). (c) Time-course measurements of tumor volumes every three or two days. (d) Measurement of tumor weight after treatment. (e) Time-course measurements of body weights every three days. All data are presented as the mean ± SD (*n* = 5). ∗∗*P* < 0.01; ∗∗∗*P* < 0.001; ∗∗∗∗*P* < 0.0001.

We next used a nude mouse subcutaneous xenograft model to evaluate the in vivo therapeutic effect of the combination treatment. For this, nude mice with GBM explants were administered vehicle, ZSTK474, TMZ, or the combination for two weeks, and mouse body weight and tumor volume were measured everyday. After the mice were sacrificed, the tumors were excised for weight measurement. As shown in Figures 6(a)–6(d), ZSTK474 or TMZ as single agents exhibited significant inhibition of tumor growth. Furthermore, the combination treatment nearly completely suppressed tumor growth, with an over 90% reduction in both tumor volume and weight. Additionally, no obvious toxicity of the monotherapy and combination treatment was observed according to the body weight during the 14-day treatment (Figure 6(e)).

## 4. Discussion

GBM is an incurable, malignant tumor of the central nervous system [32]. The introduction of the alkylating agent TMZ to the standard therapy has prolonged the survival time of GBM patients to a certain extent. However, a number of GBM cases lack a response to TMZ therapy [3], and the need for more effective treatment modalities that increase the sensitivity of TMZ to GBM is overwhelming. The mechanism of TMZ resistance is rather intricate, and one of the leading causes is the upregulation of DNA repair pathways that reverse therapy-induced DNA damage, which makes targeting the DDR a potential strategy to sensitize tumor cells to treatment. In this study, we report that the PI3K inhibitor ZSTK474 accelerated TMZ-induced DSBs by suppressing the HR repair process and sensitizing GBM cells to TMZ.

We first demonstrated that TMZ treatment induced the most lethal lesion DSBs in GBM SF295 and U87 cells, accompanied by upregulation of HR and NHEJ repair proteins. ATM is a key factor that recognizes DNA damage to initiate HR repair, and downstream molecules, including BCRA1, RPA, and RAD51, are assembled at the DNA break site to complete the repair process [33]. In addition, Ku70/80 and DNA-PKcs are all involved in the NHEJ machinery in response to DSBs [34]. Therefore, we can infer that TMZ kills tumor cells and simultaneously causes DDR and activates HR and NHEJ repair; thus, inhibiting the two repair pathways may enhance the antitumor effect of TMZ. In addition, we found that TMZ resulted in the compensatory activation of the PI3K signaling pathway. The PI3K catalytic subunits PI3K-p110*α* and PI3K-p110*β*, as well as the activities of the downstream key molecules Akt and mTOR, were all elevated after incubation with TMZ. Moreover, some studies also show that endogenous Akt activity can be activated in response to clinically relevant concentrations of TMZ [12]. Since studies indicate that blockade of the PI3K pathway could enhance sensitivity to DNA-damaging treatments by suppressing NHEJ and HR repair [35], we hypothesized that the pan-PI3K inhibitor ZSTK474 may potentiate the cytotoxicity of TMZ in GBM cells.

We combined the use of ZSTK474 and TMZ in three GBM cell lines and in a subcutaneous xenograft model. As expected, the two drugs synergistically inhibited GBM cell growth in vitro and in vivo. Treatment with other PI3K inhibitors (BYL719 and TGX221) could also mimic the sensitization mediated by ZSTK474, suggesting that blockade of the PI3K pathway is effective in sensitizing tumor cells to TMZ. We next analyzed the effect of the combination treatment on cell apoptosis and cell cycle distribution. The combined use of ZSTK474 and TMZ exhibited a stronger apoptosis-inducing effect than either drug alone. Cell cycle analysis indicated that TMZ alone arrested the cell cycle in G2/M phase, but when combined with ZSTK474, this effect was attenuated. It was demonstrated that TMZ preferentially induces cells to arrest in the G2/M phase to repair damaged DNA [36]. In particular, HR repair can only work if cells are damaged in the S/G2 phase of the cell cycle [14]. Therefore, ZSTK474 inhibited DNA damage repair and eventually led to apoptosis, which might be related to increased G0/G1 phase or the attenuation of G2/M checkpoint arrest caused by TMZ.

We also found that ZSTK474 further increased TMZ-induced DSBs. It is well known that HR and NHEJ are two major pathways to repair DSBs [37], so we examined whether ZSTK474 enhanced the effect of TMZ by targeting HR and/or NHEJ. The results revealed that ZSTK474 blocked HR rather than NHEJ repair efficiency. Although the PI3K/mTOR inhibitors PKI-587, NVP-BEZ235, and omipalisib were demonstrated to suppress NHEJ to sensitize cancer cells to radiotherapy and/or chemotherapy [38–41], ZSTK474 did not exert the same effect. HR repair is a high-fidelity and template-dependent repair that comprises a number of interrelated pathways that function in the repair of DNA DSBs [42]. We found that the combined use of ZSTK474 and TMZ could inhibit a series of key HR repair factors, including the phosphorylation of ATM and the expression of NBS1, BRCA1, BRCA2, and RAD51, compared with TMZ alone. Following DNA DSBs, ATM is primarily activated through interactions with NBS1 of the MRN complex [43], which leads to the phosphorylation of the downstream targets to complete the repair process. The BRCA1 and BRCA2 proteins function in recombination with the recombinase RAD51, which is necessary for HR [42]. Furthermore, mounting evidence has shown that PI3K inhibition can induce HR deficiency by directly or indirectly regulating HR repair molecules. Chiang et al. reported that c-Myc promoted the transcription of the NBS1 gene involved in DNA DSB repair [44]. C-Myc is also known as a downstream effector of the PI3K/Akt pathway [45]. In addition, PI3K-p110*β* was proven to regulate the binding of the NBS1 sensor protein to damaged DNA [16]. As NBS1 can form a complex with Mre11 and Rad50, which will activate ATM at DNA break sites [46], so PI3K inhibitors might reduce the phosphorylation level of ATM through downregulation of NBS1. Here, we showed that PI3K-p110*β*, p-Akt, p-mTOR, and c-Myc were all downregulated after combination treatment compared to TMZ alone. Moreover, ZSTK474 decreased BRCA1/2 expression, which might be attributed to the activation of ETS transcription factors that are involved in ERK-dependent BRCA1/2 downregulation [28]. Since Ibrahim and Juvekar et al. for the first time reported that blockage of PI3K/Akt pathway could result in HR deficiency by reducing BRCA1/2 expression and Rad51 foci formation in breast cancer [28, 47], a number of clinical trials and preclinical research combining PI3K inhibitors with PARP inhibitors were performed on HR proficient cancers [48–51]. Therefore, as a PI3K inhibitor, ZSTK474 has potential to be used as a sensitizer in combination with DNA-damaging treatments, including chemotherapy agents and radiotherapy.

## 5. Conclusion

**Figure 7:**
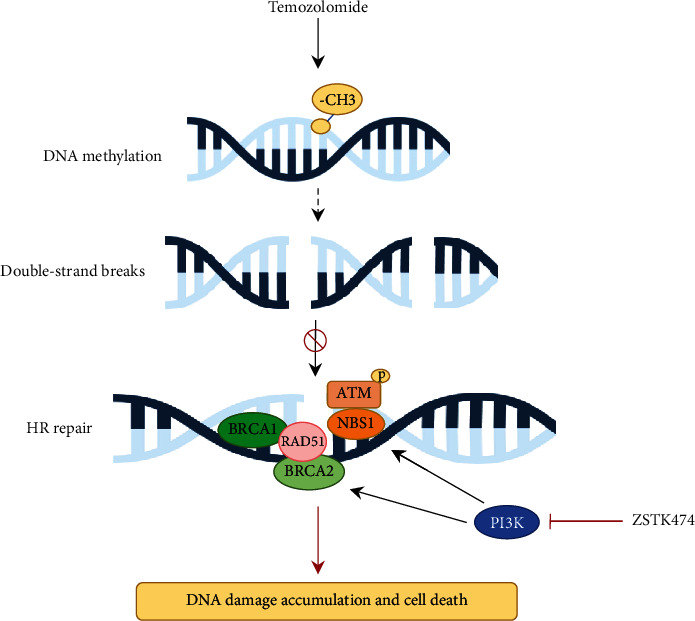
Schematic diagram of the mechanism of ZSTK474 sensitizes GBM to TMZ treatment.

In summary, we reported that the PI3K inhibitor ZSTK474 and TMZ synergistically inhibited GBM cell growth and induced apoptosis and DNA damage in GBM cells. PI3K inhibition by ZSTK474 caused “HR deficiency” that sensitized GBM cells to TMZ treatment (Figure 7). Our study suggests that the combination of ZSTK474 and TMZ appears to be a promising regimen for the treatment of GBM.

## Figures and Tables

**Table 1:** Combination indexes (CI) of PI3K inhibitors and TMZ in GBM cells.

Drug combination	Cells	*r*	CI values		
			ED50	ED75	ED90
ZSTK474 and TMZ	SF295	0.995	0.07	0.14	0.27
	U87	0.982	0.19	0.08	0.06
	U251	0.919	0.17	0.08	0.03
BYL719 and TMZ	SF295	0.940	0.48	0.53	0.58
	U87	0.989	0.06	0.12	0.22
TGX221 and TMZ	SF295	0.991	0.05	0.07	0.10
	U87	0.953	0.18	0.28	0.46

## Data Availability

The data used to support the findings of this study are available from the corresponding author upon request.

## Supplementary Materials

Supplementary Materials Supplementary Figure 1: the effect of the combination of ZSTK474 and TMZ on the cell cycle distribution of GBM SF295 cells.
