# Supplementary material for: ZSTK474 Sensitizes Glioblastoma to Temozolomide by Blocking Homologous Recombination Repair
Source: Biomed Res Int. 2022 Jul 13;2022:8568528. doi: 10.1155/2022/8568528 (PMC9300311; doi:10.1155/2022/8568528)
Supplement: Supplementary Materials — Supplementary Figure 1: the effect of the combination of ZSTK474 and TMZ on the cell cycle distribution of GBM SF295 cells. [file 8568528.f1.pdf]

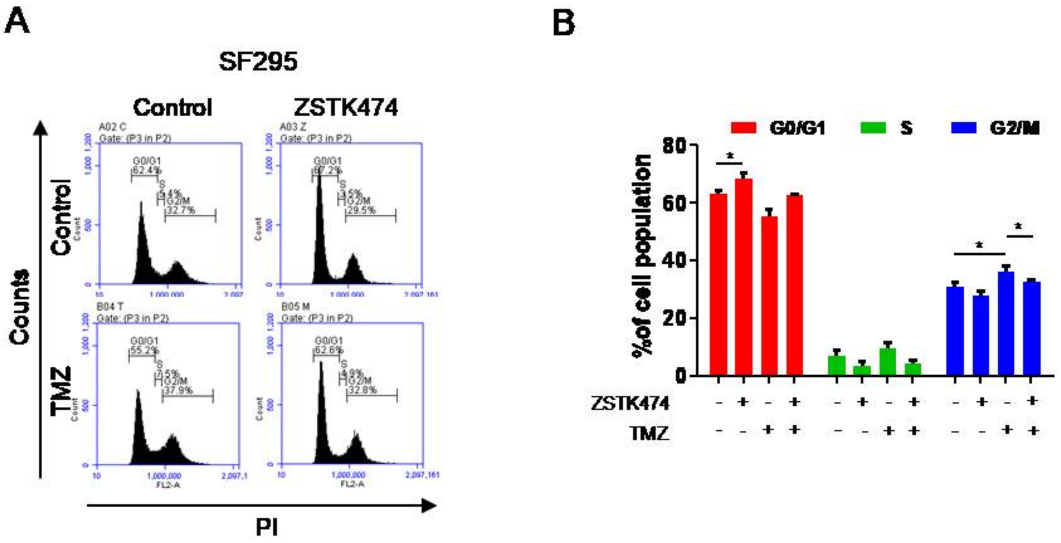

Suppl figure 1. The effect of the combination of ZSTK474 and TMZ on the cell cycle distribution of GBM SF295 cells. SF295 cells were treated ZSTK474 (0.4 μM and TMZ (120 μM) as single agents or in combination for 48 h. (A) The cells were then subjected to PI staining and flow cytometric analysis of cell cycle distribution. (B) FACS quantification of cell percentages in G1, S and G2/M phases. All data are presented as the mean ± SD (n=3). *P < 0.05.
